# *In vitro* efficacy of 21 dual antimicrobial combinations comprising novel and currently recommended combinations for treatment of drug resistant gonorrhoea in future era

**DOI:** 10.1371/journal.pone.0193678

**Published:** 2018-03-06

**Authors:** Vikram Singh, Manju Bala, Aradhana Bhargava, Monika Kakran, Ravi Bhatnagar

**Affiliations:** 1 Apex Regional STI Training, Research and Reference Laboratory, VMMC & Safdarjung Hospital, New Delhi, India; 2 SunRise University, Rajasthan, India; Emory University School of Medicine, UNITED STATES

## Abstract

**Background:**

Recent WHO guidelines recommend dual therapy with ceftriaxone or cefixime plus azithromycin for gonorrhea. Azithromycin in combination with gentamicin or spectinomycin has been recommended in treatment failure cases. Due to emergence of multi-drug resistant (MDR) and extensively-drug resistant (XDR) *Neisseria gonorrhoeae* strains, it is important to look for efficacy of these combinations and also of others that might be used in future. Therefore, we aimed to evaluate *in vitro* synergy of 21 dual combinations including current and alternative WHO recommended treatment regimens and other dual combinations.

**Methods and findings:**

The potential utility of *in-vitro* interactions of 21 combinations was investigated against 95 *N*. *gonorrhoeae* strains including 79 MDR and one XDR strain collected during March 2013 to July 2017 and fractional inhibitory concentration index (FICI) was calculated. These 21 combinations comprised of two WHO currently recommended (cefixime+azithromycin, ceftriaxone+azithromycin); two WHO recommended in treatment failure cases (azithromycin+gentamicin, spectinomycin+azithromycin) and other 17 combinations.

**Results:**

FICI of the four WHO recommended antimicrobial combinations were higher (>1.0) than the five novel combinationbreeds (FICI range 0.603–0.951) in the study i.e. gentamicin+ertapenem, moxifloxacin+ertapenem, spectinomycin+ertapenem, azithromycin+ moxifloxacin, cefixime+gentamicin. No antagonistic effect of the above four WHO recommended combinations except spectinomycin+azithromycin (FICI = 4.25) was observed for the XDR strain. Out of above five novel combinations, four combinations produced high synergistic effects in overall 95 strains and also for the XDR strain with FICI of 0.13 to 0.38. Antagonistic effects varying from 3.2 to 12.6% were observed for 10 out of 21 tested combinations (azithromycin in combination with gentamicin and spectinomycin; ceftriaxone with moxifloxacin, gentamicin, spectinomycin and ertapenem; spectinomycin with moxifloxacin and gentamicin; cefixime and gentamicin combination with moxifloxacin).

**Conclusion:**

WHO recommended cefixime+azithromycin, ceftriaxone+azithromycin combinations having no antagonism indicates their continuing clinical utility. Highest antagonism without any synergistic effect for the WHO recommended spectinomycin+azithromycin in treatment failure cases suggests that this combination should be evaluated further both *in vitro* and *in vivo*. Highest synergistic or additive effect without any antagonistic effect of the above five novel combinations suggests that these may be recommended for treatment in future.

## Introduction

Gonorrhea is a major public health problem with serious health, social and economic consequences worldwide despite ongoing efforts towards its treatment and control by many international agencies such as the World Health Organization (WHO) and the Centers for Disease Control and Prevention (CDC) [1,2]. Emergence of multidrug-resistant *Neisseria gonorrhoeae* (MDR-NG) and extensively drug-resistant *N*. *gonorrhoeae* (XDR-NG) strains with high-level resistance to the expanded-spectrum cephalosporins (ESCs) and azithromycin has been reported from different countries around the world [3–8]. Therefore, the increase in antimicrobial resistance (AMR) towards drugs used to treat gonorrhoea is reaching a critical stage. This coupled with lack of new treatments in the pipeline, raises the threat that the *N*. *gonorrhoeae* may become a widespread untreatable ‘superbug’ in the near future [6].

In India and many other developing countries, under syndromic management of urethral and cervical discharge syndrome, cefixime (400mg) plus azithromycin (1g) orally is recommended for treatment of gonococcal and chlamydial infections [9]. The emergence of decreased susceptibility or resistance to ESCs and azithromycin in India and other countries of South-East Asia region threatens the future utility and could be a major public health challenge to the syndromic management [5].

The crisis of AMR and treatment failure of gonorrhoea with last available antimicrobials has led to recommendations for new dual antimicrobial therapies. Therefore, the current WHO and CDC guidelines for treatment of symptomatic and asymptomatic urogenital, anorectal, or oropharyngeal gonorrhea recommend dual antibiotic therapy with ceftriaxone 250 mg intramuscular plus azithromycin 1 g orally as a single dose or cefixime 400 mg oral plus azithromycin 1 g orally as a single dose [10,11]. However, in 2016, one patient with pharyngeal gonorrhoea considered to have treatment failure with currently recommended dual therapy was reported from United Kingdom because the post-treatment isolate was resistant to ceftriaxone and azithromycin [12]. Due to the loss of effective and readily available treatment options, there is an immediate need to find out the new therapeutic options for treating gonococcus infections and reduce the increased levels of AMR.

Antimicrobial combination therapy may be the only option in near future for cases of drug resistant gonococcal strains. Combination therapy due to synergistic or additive effect of the combined antimicrobials may be able to delay the emergence of ESC resistant *N*. *gonorrhoeae* isolates [13]. While additional combinations of existing antibiotics are currently being evaluated, there are no new antibiotics in late development. As such, even new classes of antibiotics may only provide a short-term solution given the ability of *N*. *gonorrhoeae* to rapidly develop resistance. Therefore, WHO’s Global Action Plan to control the spread and impact of antimicrobial resistance in *N*. *gonorrhoeae* published in 2012 highlights the need for evaluation of dual antimicrobial therapy as one of the primary area of interest for operational research [14].

We recently observed in a study on synergy testing that *in vitro* activity of gentamicin was enhanced against MDR and XDR *N*. *gonorrhoeae* strains in combination with ertapenem and cefixime [15].

Some of the studies have earlier investigated *in vitro* synergy of WHO and CDC recommended combinations and the reports on their efficacy against *N*. *gonorrhoeae* were conflicting [16–18]. Moreover, these studies were conducted on limited number of *N*. *gonorrhoeae* isolates using fewer number of antimicrobial combinations i.e. 4 combinations against 25 isolates in Japan, 3 combinations against 64 isolates in London, 10 combinations against 28 isolates in United States [16–18]. Hence in this study, we explored the synergistic, additive, indifferent or antagonistic effects of 21 antimicrobial combinations on 95 *N*. *gonorrhoeae* strains as follows:

1. two antimicrobial combinations currently recommended by the WHO for management of gonococcal infection**s** i.e. cefixime plus azithromycin and ceftriaxone plus azithromycin;
2. two antimicrobial combinations recommended in cases of treatment failure with a WHO-recommended dual therapy i.e. azithromycin plus gentamicin and spectinomycin plus azithromycin combination
3. other 17 antibiotic combinations with cefixime, ceftriaxone, spectinomycin, azithromycin, gentamicin, moxifloxacin and ertapenem to find out the novel antimicrobial combinations useful for treatment of the MDR or XDR infections and overcome the crisis of AMR of *N*. *gonorrhoeae* in near future.

## Methods

Ethical approval was obtained from the Institute Ethical Committee of Safdarjung Hospital and VMMC. Approval number: VMMC/SJH/PROJECT/2013/30-1/12 dated 26 February 2013.

During the study period of March 2013 to July 2017, a total of 138 consecutive *N*. *gonorrhoeae* isolates were obtained using standard procedures for isolation and identification [8,19]. Written informed consent was taken from the participants. Minimum inhibitory concentration (MIC) of all the *N*. *gonorrhoeae* isolates was determined for penicillin, tetracycline, ciprofloxacin, cefixime, ceftriaxone, spectinomycin, azithromycin, gentamicin, moxifloxacin and ertapenem by the Etest method using the manufacturer’s (bioMerieux SA, 69280 Marcy I’Etoile, France)) instructions [20,21]. Latest interpretive criteria were used for characterisation of strains as susceptible, less susceptible, resistant, MDR and XDR [3,8, 15,19,22].

Out of 138 clinical isolates of *N*. *gonorrhoeae*, 81 isolates were selected for antimicrobial combination testing. Dual antimicrobial testing was also carried out for 10 *N*. *gonorrhoeae* reference strains [nine WHO reference strains (WHO C, F, G, K, L, M, N, O, P) and one ATCC 49226] and four quality assurance strains received during external quality assurance scheme (EQAS) testing [15]. *N*. *gonorrhoeae* isolates and reference strains were preserved by lyophilisation and were also stored in 20% glycerol broth at -70°C.

Dual antimicrobial synergy testing was performed using seven antimicrobials (cefixime, ceftriaxone, spectinomycin, azithromycin, gentamicin, moxifloxacin and ertapenem) which resulted in 21 different antibiotic combinations as follows: cefixime+spectinomycin, cefixime+azithromycin, cefixime+gentamicin, cefixime+moxifloxacin, cefixime+ertapenem, ceftriaxone+cefixime, ceftriaxone+spectinomycin, ceftriaxone+azithromycin, ceftriaxone+ gentamicin, ceftriaxone+moxifloxacin, ceftriaxone+ertapenem, spectinomycin+azithromycin, spectinomycin+gentamicin, spectinomycin+moxifloxacin, spectinomycin+ertapenem, azithromycin+gentamicin, azithromycin+moxifloxacin, azithromycin+ertapenem, gentamicin +moxifloxacin, gentamicin+ertapenem, moxifloxacin+ertapenem. Etest MIC fixed ratio method was used for synergy testing [23]. Briefly, an Etest strip of antimicrobial A was applied on an inoculated agar plate and incubated for one hour at room temperature in 5% CO_2_ enriched environment. Etest strip of antimicrobial A was removed and antimicrobial B strip was placed at the same location of antimicrobial A. Culture plate was incubated in candle jar with 5% CO_2_ enriched environment at 36°C for 18–24 hours and the MIC was recorded.

To evaluate the synergistic, additive, indifference or antagonistic effect of the combination, the fractional inhibitory concentration index (FICI) was calculated for each antibiotic combination. Detailed methodology for calculation of FICI has been described previously [15]. Briefly, FICI was calculated as follows:

$$\text{FICI}={\text{MIC}}_{\text{AB}}/{\text{MIC}}_{\text{A}}+{\text{MIC}}_{\text{BA}}/{\text{MIC}}_{\text{B}},$$

Where, MIC_AB_ = MIC of A in the presence of drug B; MIC_A_ = MIC of drug A alone; MIC_BA_ = MIC of B in the presence of drug A; MIC_B_ = MIC of drug B alone.

Synergy was defined as a FICI of ≤0.5; No interaction: >0.5 to 4.0 (additive: > 0.5 to ≤1.0; indifference: FIC >1.0 to ≤4.0) and antagonism by FICI of > 4.0 [23, 24]. Every tenth clinical isolate and two quality assurance strains were tested in duplicate for all the antibiotics to check the reproducibility of the testing method. The quality control of the MIC testing was ensured by using the WHO reference strains namely WHO C, WHO F-P and ATCC 49226 for internal quality control and by participating in EQAS every year conducted by WHO Collaborating Centre for STD and HIV, Sydney, Australia [19].

### Statistical analysis

The mean of MICs and FICIs were calculated as geometric means. The statistical significance of comparison of the MICs of antimicrobial in alone and in combination was analyzed by applying the nonparametric Mann-Whitney’s *U*-test and *p* value was determined. The *p* values of <0.05 were considered as statistically significant.

## Results

### Phenotypic antibiogram of *N*. *gonorrhoeae* strains tested by antimicrobial combination testing

In this study, antimicrobial combination testing was performed on a total of 95 *N*. *gonorrhoeae* strains. Out of the 95 strains, 42, 22, 4, 3, 1, 1 and 6 strains were penicillinase-producing *N*. *gonorrhoeae* (PPNG) + tetracycline resistant *N*. *gonorrhoeae* (TRNG) + quinolone resistant *N*. *gonorrhoeae* (QRNG), PPNG + QRNG, TRNG + QRNG, PPNG + QRNG + ceftriaxone decreased susceptibility (DS), PPNG + spectinomycin resistant (R), PPNG + QRNG + azithromycin R and QRNG + azithromycin R + penicillin/tetracycline less susceptible (LS) respectively and in total 79 strains met the criteria of MDR definition. One strain was under XDR category and it was resistant to ESCs (MIC of ceftriaxone = 1mg/L and cefixime = 4 mg/L). This strain was received from WHO collaborating centre, Sydney in 2014 EQAS panel. Of the remaining 15 isolates, which were neither MDR nor XDR, 4 isolates were QRNG + chromosomally mediated resistant *N*. *gonorrhoeae* (CMRNG) to penicillin/tetracycline + ceftriaxone DS, 1 was QRNG+CMRNG to penicillin+ tetracycline LS, 4 QRNG + penicillin/tetracycline less LS, 2 azithromycin R+ penicillin/tetracycline LS, 1 CMRNG penicillin+tetracycline LS and 3 were penicillin/tetracycline LS.

The results of all the tested strains on repeat testing for different antimicrobial combinations remained in the same interpretive category determining the reproducibility of the technique. Interactions of 1995 antimicrobial combination tests evaluated for 95 *N*. *gonorrhoeae* strains are shown in Tables 1 and 2. MICs of all the antibiotics individually, MIC_AB_, MIC_BA_ and FICI against all the 95 strains are depicted in excel data sheets in S1 Table.

**Table 1:** Synergistic effects of four WHO currently recommended and five novel antimicrobial combinations with geometric mean of MICs alone and in combination with FICI values.

Combination testing results	WHO currently recommended combinations		WHO combinations recommended in treatment failure cases		Combinations for future clinical use				
	IX+AZ	TX+AZ	AZ+GM	SC+AZ	MX+ETP	GM+ETP	SC+ETP	AZ+MX	IX+GM
**Synergy n (%)**	7 (7.4)	5 (5.2)	11 (11.6)	0	36 (37.9)	30 (31.6)	22 (23.2)	16 (16.8)	10 (10.5)
**Additive n (%)**	14 (14.7)	26 (27.4)	22 (23.2)	27 (28.4)	30 (31.6)	35 (36.8)	29 (30.5)	26 (27.4)	20 (21.1)
**Indifference n (%)**	74 (77.9)	64 (67.4)	56 (58.9)	56 (59.0)	29 (30.5)	30 (31.6)	44 (46.3)	53 (55.8)	65 (68.4)
**Antagonistic n (%)**	0	0	6 (6.3)	12 (12.6)	0	0	0	0	0
**MIC**_**A**_ **Alone**	0.018	0.009	0.203	9.153	1.075	2.436	9.153	0.203	0.018
**MIC**_**AB**_ **Combination (p value)**	0.016 (0.057)	0.007 (0.22)	0.196 (0.624)	1.015 (<0.0001)	0.008 (<0.0001)	0.092 (<0.0001)	0.242 (<0.0001)	0.150 (0.057)	0.016 (0.002)
**MIC**_**B**_ **Alone**	0.203	0.203	2.436	0.203	0.011	0.011	0.011	1.075	2.436
**MIC**_**BA**_ **Combination (p value)**	0.016 (<0.0001)	0.043 (<0.0001)	0.348 (<0.0001)	0.237 (0.177)	0.007 (<0.0001)	0.006 (<0.0001)	0.008 (0.0147)	0.023 (<0.0001)	0.031 (<0.0001)
**FICI**	1.039	1.192	1.257	1.442	0.732	0.603	0.753	0.951	0.855

n, Number; IX, Cefixime; AZ, Azithromycin; TX, Ceftriaxone; GM, Gentamicin; SC, Spectinomycin; MX, Moxifloxacin; ETP, Ertapenem.

**Table 2:** Synergistic effects of other 12 antimicrobial combinations with geometric mean of MICs alone and in combination with FICI values.

Combination testing results	Antimicrobial combinations											
	AZ+ETP	IX+SC	IX+ETP	TX+IX	TX+MX	TX+GM	TX+SC	TX+ETP	SC+MX	IX+MX	GM+MX	SC+GM
**Synergy n (%)**	7 (7.4)	7 (7.4)	6 (6.3)	2 (2.1)	20 (21)	14 (14.7)	12 (12.6)	12 (12.6)	10 (10.5)	9 (9.5)	7 (7.4)	6 (6.3)
**Additive n (%)**	40 (42.1)	38 (40)	16 (16.8)	13 (13.7)	34 (35.8)	26 (27.4)	26 (27.4)	38 (40)	25 (26.3)	64 (67.3)	22 (23.1)	21 (22.1)
**Indifference n (%)**	48 (50.5)	50 (52.6)	73 (76.9)	80 (84.2)	36 (37.9)	49 (51.6)	52 (54.7)	42 (44.2)	54 (56.9)	19 (20)	57 (60)	65 (68.4)
**Antagonistic n (%)**	0	0	0	0	5 (5.3)	6 (6.3)	5 (5.3)	3 (3.2)	6 (6.3)	3 (3.2)	9 (9.5)	3 (3.2)
**MIC**_**A**_ **Alone**	0.203	0.018	0.018	0.009	0.009	0.009	0.009	0.009	9.153	0.018	2.436	9.153
**MIC**_**AB**_ **Combination**	0.049	0.016	0.015	0.003	0.008	0.008	0.008	0.005	5.358	0.016	1.960	4.121
**MIC**_**B**_ **Alone**	0.011	9.153	0.011	9.153	1.075	2.436	9.153	0.011	1.075	1.075	1.075	2.436
**MIC**_**BA**_ **Combination**	0.006	0.072	0.002	0.017	0.007	0.091	0.197	0.004	0.183	0.002	0.148	1.951
**FICI**	0.976	0.926	1.092	1.313	0.971	0.977	0.955	1.039	1.065	0.980	1.227	1.384

n, Number; AZ, Azithromycin; ETP, Ertapenem; IX, Cefixime; SC, Spectinomycin; TX, Ceftriaxone MX, Moxifloxacin; GM, Gentamicin.

combinations with moxifloxacin and gentamicin; cefixime plus moxifloxacin and gentamicin plus moxifloxacin (Table 2).

### I. Efficacy of two antimicrobial combinations currently recommended by the WHO for management of gonococcal infections

#### Cefixime and azithromycin combination

Synergistic or additive effect of cefixime plus azithromycin combination was observed only in 22.1% of strains. Although this combination did not display any antagonistic effect, indifference effect was seen in 77.9% of strains (Table 1). Cefixime when tested alone, MICs ranged between 0.012 mg/L to 4 mg/L and geometric mean of MICs was 0.018 mg/L, whereas in combination with azithromycin, MICs range decreased insignificantly (0.012 mg/L to 0.25 mg/L) with mean value of 0.016mg/L. The geometric mean FICI value for cefixime plus azithromycin was 1.039, representing indifference effect (Table 1).

#### Ceftriaxone plus azithromycin combination

Ceftriaxone plus azithromycin combination displayed either synergistic (5.2%), additive (27.4%) or indifference (67.4%) effect in 100% of strains without any antagonistic effect (Table 1). The MICs of ceftriaxone as alone ranged from 0.0015mg/L to 2 mg/L and geometric mean MIC value was 0.009 mg/L. With ceftriaxone plus azithromycin combination, there was reduction in the geometric mean MIC to 0.007 mg/L and MICs ranged from 0.0015 mg/L to 0.125 mg/L. The geometric mean of FICI depicted indifference effects for the paired use of ceftriaxone and azithromycin (Table 1).

Five strains showed FICI value under ≤ 0.5 (synergistic effect) as 0.13, 0.38, 0.38, 0.42, 0.50. MICs of four out of these five isolates decreased by two doubling dilutions, while of fifth isolate decreased by one doubling dilution.

### II. Efficacy of two antimicrobial combinations recommended by WHO in cases of treatment failure with the recommended dual therapy

#### Azithromycin plus gentamicin combination

Either synergistic, additive or indifference effect was found in 93.7% of strains in azithromycin plus gentamicin combination (Table 1). The MICs of azithromycin in alone ranged from 0.016 mg/L to 16.0 mg/L with mean MIC value as 0.203 but with combination of gentamicin it ranged from 0.012 mg/L to 3.0 mg/L and mean MIC value insignificantly decreased to 0.196 mg/L. Gentamicin mean MIC was 2.436 mg/L which significantly decreased to 0.348 mg/L with the combination effects of azithromycin (Table 1).

#### Spectinomycin and azithromycin combination

The spectinomycin plus azithromycin combination produced highest (12.6%) antagonistic effects without any synergistic effects (Table 1). The geometric mean of MICs of spectinomycin alone was 9.153 mg/L which significantly decreased to 1.015 mg/L in combination with azithromycin, whereas azithromycin geometric mean of MICs alone was 0.203 which insignificantly increased to 0.237 mg/L in combination with spectinomycin. The geometric mean of FICI for this combination was 1.442 which demonstrated as indifference (Table 1).

FICI of the above four recommended combinations (cefixime plus azithromycin, ceftriaxone plus azithromycin, azithromycin plus gentamicin, spectinomycin plus azithromycin) were 2.13, 2.03, 2.06 and 4.25 respectively for the highly resistant strain (QA2).

### III. Efficacy of other 17 antibiotic combinations tested in the study

#### IIIA. Five novel antimicrobial combination breeds in the study

The results of five novel antimicrobial combinations which presented synergistic, additive or indifference effect without any antagonism are summarised in Table 1. Highest *in vitro* synergistic or additive effect was observed with moxifloxacin plus ertapenem combination, without any antagonistic effects. The MICs of moxifloxacin as alone ranged from 0.004 mg/L to 16 mg/L with geometric mean MIC as 1.075 mg/L whereas in combination effects of ertapenem MICs range decreased (0.001 mg/L to 1.00 mg/L) with geometric mean MIC as 0.008 mg/L (Table 1). The MICs of ertapenem when used alone ranged from 0.002 mg/L to 0.125 mg/L and geometric mean of MICs was 0.011 mg/L, whereas in combination with moxifloxacin, the MICs range of ertapenem decreased to 0.0015–0.064 mg/L with mean value of 0.007 mg/L. The mean FICI value of this combination was 0.732 depicting additive effects (Table 1).

The second highest synergic/additive effect was observed in combination of gentamicin plus ertapenem (Table 1). The mean FICI value was 0.603 for this combination representing additive effect. The MICs of gentamicin in alone ranged from 1.0 mg/L to 8.0 mg/L and geometric mean value of all MICs was 2.436 mg/L but in combination of ertapenem, range came down (0.012 mg/L to 2.0 mg/L) and geometric mean MIC value significantly decreased to 0.092 mg/L Ertapenem when tested as single drug, MICs ranged from 0.002 mg/L to 0.125 mg/L and geometric mean MIC was 0.011 mg/L, whereas in combination with gentamicin, the MICs range of ertapenem was decreased to 0.0015 mg/L to 0.064 mg/L with mean value of 0.006 mg/L (Table 1).

Spectinomycin plus ertapenem combination revealed third highest efficacy. Spectinomycin MICs ranged from 2 to 1536 mg/L with geometric mean of MICs being 9.153 mg/L which significantly decreased to 0.242 mg/L in combination of ertapenem. The geometric mean of FICI for spectinomycin plus ertapenem combination was 0.753 for all isolates and was interpreted as additive effect (Table 1).

Azithromycin and moxifloxacin combination presented with 44.2% synergistic or additive, effects without any antagonism and mean FICI value was 0.951. The MICs of azithromycin ranged from 0.016 mg/L to 16 mg/L and geometric mean of MICs was 0.203 mg/L which decreased to 0.150 mg/L (*p* value = 0.057) in combination with moxifloxacin (Table 1).

Cefixime along with gentamicin displayed synergistic or additive effect against 31.6% of strains without any antagonistic effects (Table 1). The geometric mean MIC of cefixime was 0.018 mg/L in alone, which significantly decreased to 0.016 mg/L with combination of gentamicin. The MICs range for cefixime was 0.012 mg/L to 4.0 mg/L when tested alone, whereas with combination of gentamicin it ranged between 0.012 mg/L to 0.75 mg/L. The mean MIC value of gentamicin was 2.436 mg/L which significantly decreased to 0.031 mg/L (*p* value <0.0001) with cefixime combination. The geometric mean FICI value indicated additive effects with dual testing of cefixime with gentamicin.

Out of above five novel combinations, four combinations also produced effective synergistic effects for the XDR strain (QA2/14) i.e. the combinations of moxifloxacin plus ertapenem (FICI = 0.13), gentamicin plus ertapenem (FICI = 0.13), spectinomycin plus ertapenem (FICI = 0.27) and cefixime plus gentamicin (FICI = 0.38). Only azithromycin plus moxifloxacin did not demonstrate synergistic effects (FICI = 2.02) in this strain.

#### III B. Synergistic effects of the remaining 12 antibiotic combinations

The combinations of azithromycin plus ertapenem, cefixime plus spectinomycin, cefixime plus ertapenem and ceftriaxone plus cefixime had synergistic/additive effects in 49.5%, 47.4%, 23.1% and 15.8% of strains without any antagonism and the mean FICI values were 0.976, 0.926, 1.092 and 1.313 respectively representing additive or indifference effect (Table 2). The antagonistic effects ranging from 3.2% to 9.5% were observed to combinations of ceftriaxone with moxifloxacin, gentamicin, spectinomycin and ertapenem; spectinomycin.

## Discussion

Most promising approach to combat gonococcal resistant bacteria and to delay the onset of treatment failures is dual therapy. Presently, ESCs (ceftriaxone or cefixime) combined with azithromycin remains a satisfactory option for the first-line treatment of gonorrhoea. But as resistance to ESCs and azithromycin continues to rise, it poses a significant threat to the currently recommended dual therapy. Therefore, in the present study, *in vitro* efficacy of existing and new antimicrobial combinations for future use was investigated.

In the present study, WHO recommended combination of cefixime+azithromycin and ceftriaxone+azithromycin displayed synergistic or additive effect with no antagonism. Some of the previous studies have observed *in vitro* synergy between ceftriaxone or cefixime and azithromycin [16, 25,26] but no synergy was observed for these two combinations in other studies [17,18,27,28]. Our results are comparable with the Japanese study [16], where 32% synergy was observed for cefixime plus azithromycin. In the Swedish study, 63% and 34% synergy was reported for cefixime+azithromycin and ceftriaxone+azithromycin combination respectively. Etest method was used in our study for combination testing while both the Etest and agar dilution method were used in the USA and Netherlands study [18, 27]. Results were comparable by both the methods in these two studies. Only agar dilution method was used in the London study [17] and the mean FICI values were more than 1.0 for both the above combinations in our and London study indicating that the difference between methods did not lead to a difference in interpretation of synergy. No antagonistic effect was observed with both the combinations currently recommended by WHO in our study and in any of the earlier study indicating that this combination has clinical utility [16–18, 25–27].

Gentamicin is being used in Malawi for more than 20 years for treatment of gonorrhea and it had high *in vitro* efficacy against MDR stains in a recent study [19,29]. Gentamicin is currently recommended by the WHO in combination with azithromycin in cases of treatment failure [10]. Only three studies have earlier determined the *in vitro* interaction of gentamicin+ azithromycin [16,17,27]. In the UK study, a mean FICI of 1.7 was observed for all gonococcal isolates depicting additive/indifference with the antimicrobial combination of gentamicin with azithromycin and is comparable with FICI value of 1.7 and 1.2 found in the Netherlands and our study [17, 27]. While in a study from Japan in 2013, mean FICI was 0.83 with this combination [16]. In a clinical randomized trial by CDC in the USA, 240 mg gentamicin intramuscularly and 2 g of azithromycin was found to be highly effective for treatment of mainly azithromycin susceptible *N*. *gonorrhoeae* advocating it as potential treatment regimen [30]. However, antagonism of 6.3% observed in the present study highlights that this combination should be evaluated both *in vitro* and *in vivo* against gentamicin and azithromycin resistant strains.

WHO recommended another combination in treatment failure cases (spectinomycin+azithromycin) showed no synergy and highest antagonism (12.6%) with FICI of 1.44. Findings are in contrast to that from Japan in 2013, where this combination demonstrated the additive effect with mean FICI of 0.69 [16]. Our results were comparable to the Netherlands study where FICI was 1.55.

Findings of the present study suggest five potential new candidate combinations such as gentamicin+ertapenem, moxifloxacin+ertapenem, spectinomycin+ertapenem, azithromycin+moxifloxacin, cefixime+gentamicin for treatment of MDR and XDR strains. No study except the Netherland study [27] has investigated the interactions of these five novel combinations and that also only on four *N*. *gonorrhoeae* isolates in comparison to 95 strains in our study. Moreover, the synergism of two of these five novel combinations i.e. gentamicin with ertapenem and with cefixime has been demonstrated previously by the authors [15]. In earlier study, only 7 combinations were tested, these two combinations again emerged out as novel out of 21 combinations, hence were included in the list of novel combinations. Therefore, these five combinations need to be evaluated against resistant strains in more studies.

Gentamicin and moxifloxacin in combination with ertapenem had high synergistic effect for all the strains and also the XDR strain. Our findings are in agreement with the Netherland study where no antagonism was observed with these two combinations for the ceftriaxone resistant strain [27].

FIC index for spectinomycin+ertapenem and azithromycin+moxifloxacin combination was 0.75 and 0.95 respectively and it was less than value of 1.48 and 1.31 in the Netherland study [27]. Findings of novel combination of cefixime+gentamicin (FICI = 0.85) were similar to the Netherland study i.e. mean FICI 0.97 for eleven strains tested.

The combinations of ceftriaxone plus gentamicin produced an additive effect in comparison to the USA and Netherland study where indifference effect was observed [18, 27]. Ceftriaxone plus gentamicin combination could be an active regimen against azithromycin resistant strains, although this requires clinical evidence [18].

Cefixime and ceftriaxone in combination with spectinomycin were evaluated by Lee et al. in Korea [28]. FICI values for both the combinations were either additive or indifferent, and no synergistic or antagonistic effects were found in comparison to our study.

Reason for variability of results between our study and in other published studies for some of the antimicrobial combinations could be due to the difference in strains studied in different countries or difference in the methodologies used in these studies. This has been explained in earlier studies also [17,18]. In our five novel antibiotic combination breeds all the drug pairs have different mechanism of action. While one antibiotic acts by either inhibiting protein synthesis (gentamicin, azithromycin and spectinomycin) or by inhibiting replication (moxifloxacin) the other members (ertapenem/cephalosporins) act by damaging the cell wall of the bacteria thereby enhancing the uptake of the first antibiotic. Hence, these synergistic antibiotics having different mechanism of actions, work together to produce an effect more potent than if each antibiotic were applied singly. As against this the antagonistic combination of drugs would only hinder the effect of one other.

Three best novel combinations having lowest FICI values included ertapenem. Ertapenem being a bactericidal antibiotic which inhibits cell wall synthesis; works in combination and by amplifying the effect of drugs inhibiting protein synthesis/replication there by giving lower FICI values an important finding of our study. In an earlier study, advantages of ertapenem over ceftriaxone were demonstrated for MDR or ceftriaxone resistant isolates and it was suggested that ertapenem might be effective in dual therapy [31].

## Conclusion

The findings of the study will be of interest to the international agencies formulating treatment guidelines for gonorrhoea. Further clinical studies are required to compare and implement the *in-vitro* results with clinical outcomes, especially for 9 new combinations out of 21 dual combinations tested, where no antagonism was observed. Clinical trials are also warranted for both the WHO regimens recommended in treatment failure cases as more than 5% antagonism was depicted for these two combinations. A key outcome in the study is that four recommended antibiotic combinations exhibit indifference against a strain characterized as highly resistant (QA2), which would appear to argue against the value of current combination therapy for resistant strains. However, *in vitro* results may not always correspond to a clinical effect. Gentamicin in combination with azithromycin was recommended by CDC in a clinical trial. The CDC trial was mainly against azithromycin susceptible cases. Antagonism of 6.3% for gentamicin+azithromycin combination observed in the present study highlights that this combination should be evaluated both *in vitro* and *in vivo* against large number of gentamicin and azithromycin resistant strains. However, only a few strains with resistance to gentamicin have been detected till now.

## Supporting information

S1 Table MIC values of all the antibiotics when tested alone, MIC of antibiotic A tested in combination with antibiotic B (MIC_AB_), MIC of antibiotic B tested in combination with antibiotic A (MIC_BA_) and FICI of 21 different antimicrobial combinations against *Neisseria gonorrhoeae* strains. (XLSX)

## Abbreviations

AMR: antimicrobial resistance
AZ: Azithromycin
CDC: Centre for disease control and prevention
CMRNG: chromosomally mediated resistant *N*. *gonorrhoeae*
DS: decreased susceptibility
EQAS: external quality assurance scheme
ESCs: expanded-spectrum cephalosporins
ETP: Ertapenem
FICI: fractional inhibitory concentration index
GM: Gentamicin
IX: Cefixime
LS: less susceptible
MDR: multi-drug resistant
MIC: minimum inhibitory concentration
MX: Moxifloxacin
*N*. *gonorrhoeae*: *Neisseria gonorrhoeae*
PPNG: penicillinase-producing *N*. *gonorrhoeae*
QRNG: quinolone resistant *N*. *gonorrhoeae*
R: resistant
SC: Spectinomycin
TRNG: tetracycline resistant *N*. *gonorrhoeae*
TX: Ceftriaxone
WHO: World Health Organization
XDR: extensively-drug resistant
